# miR-9 Does Not Regulate Lamin A Expression in Metastatic Cells from Lung Adenocarcinoma

**DOI:** 10.3390/ijms21051599

**Published:** 2020-02-26

**Authors:** Julien Guinde, Audrey Benoit, Diane Frankel, Stéphane Robert, Kevin Ostacolo, Nicolas Lévy, Philippe Astoul, Patrice Roll, Elise Kaspi

**Affiliations:** 1 APHM, Hôpital Nord, Department of Thoracic Oncology—Pleural Diseases—Interventional Pulmonology, CEDEX 5, 13005 Marseille, France; julien.guinde@ap-hm.fr (J.G.); pastoul@ap-hm.fr (P.A.); 2 Aix Marseille Univ, INSERM, MMG, 13005 Marseille, France; audrey.benoit@neuf.fr (A.B.); kja11@hi.is (K.O.); 3 Aix Marseille Univ, APHM, INSERM, MMG, Hôpital la Timone, Service de Biologie Cellulaire, 13005 Marseille, France; diane.frankel@univ-amu.fr (D.F.); patrice.roll@univ-amu.fr (P.R.); 4 Aix Marseille Univ, INSERM, C2VN, AMUTICYT Core facility, 13005 Marseille, France; stephane.robert@univ-amu.fr; 5 Department of Biochemistry and Molecular Biology, Biomedical Center, Faculty of Medicine, University of Iceland, 101 Reykjavik, Iceland; 6 Aix Marseille Univ, APHM, INSERM, MMG, Hôpital la Timone, Département de Génétique Médicale, 13005 Marseille, France; nicolas.levy@univ-amu.fr

**Keywords:** lamin A, microRNA-9, lung adenocarcinoma, pleural effusions

## Abstract

In lung adenocarcinoma, low lamin A expression in pleural metastatic cells has been proposed as a pejorative factor. miR-9 physiologically inhibits the expression of lamin A in neural cells and seems to be a central actor in the carcinogenesis and the metastatic process in lung cancer. Thus, it could be a good candidate to explain the reduction of lamin A expression in lung adenocarcinoma cells. miR-9 expression was analyzed in 16 pleural effusions containing metastatic cells from lung adenocarcinoma and was significantly reduced in patients from the ‘Low lamin A expression’ group compared to patients from the ‘High lamin A expression’ group. Then, carcinoma cells selection by fluorescence-activated cell sorting (FACS) was performed according to epithelial membrane antigen (EMA) expression, reflecting lamin A expression. miR-9 was underexpressed in lamin A− carcinoma cells compared to lamin A+ carcinoma cells in patients from the ‘Low lamin A expression’ group, whereas there was no difference of miR-9 expression between lamin A+ and lamin A− carcinoma cells in patients from the ‘High lamin A expression’ group. These results suggest that miR-9 does not regulate lamin A expression in metastatic cells from lung adenocarcinoma. On the contrary, miR-9 expression was shown to be reduced in lamin A-negative carcinoma cells.

## 1. Introduction

The microRNA-9 (also called miRNA-9 or miR-9) corresponding to the mature miR-9-5p is encoded by three genes in humans, namely, *microRNA-9-1*, *microRNA-9-2*, and *microRNA-9-3*, depending on their localization (chromosomes 1, 5, and 15, respectively). Firstly, miR-9 was identified as a regulator of neurogenesis [1,2,3] and is commonly associated with the carcinogenesis and the metastatic process of cancers, acting either as a tumor suppressor or as an oncogene, according to cancer type [3,4,5]. Even if miR-9 expression is repeatedly deregulated in cancer tissues, its role in tumor development and metastasis is not clear, and inconsistent results were found concerning its prognostic value [3,6].

These opposite results may be the consequence of its numerous targets and the type of cancerous tissue in which miR-9 is expressed. Several studies have demonstrated that miR-9 targets CDH1 mRNA, leading to epithelial-cadherin (E-cadherin) downregulation. This protein is involved in epithelial cells’ adhesion and polarity. The downregulation of E-cadherin expression is associated with the epithelial to mesenchymal transition (EMT) and allows the dissociation of carcinomatous cells, promoting invasion and metastatic process [7,8,9,10]. miR-9 also targets genes encoding tumor suppressor factors, such as FOXO1 (Forkhead box protein O1) and CDX2 (caudal-related homeobox 2), increasing cell growth and proliferation, respectively, in breast and gastric cancer [11,12]. miR-9 directly targets SOX7 (SRY-Box 7), which is a transcription factor involved in developmental processes and acting as a tumor suppressor in several cancers. In non-small cell lung carcinoma (NSCLC), SOX7 expression is repressed by miR-9, enhancing TGF (Transforming Growth Factor)-β1-induced NSCLC cell invasion and adhesion [13].

Conversely, miR-9 has been described as a tumor suppressor by targeting NF-kB (nuclear factor kappa light polypeptide) in gastric carcinoma [14], preventing cell proliferation in epithelial ovarian cancer [15], and inducing cell apoptosis and decreasing migration in lung adenocarcinoma cell line overexpressing miR-9 [16]. miR-9 also inhibits cell metastasis and EMT through targeting the forkhead box P2 (FOXP2) in colorectal carcinoma, and its downregulation is correlated with a poor prognosis [17]. Moreover, miR-9 is a repressor of Metastasis-Associated Lung Adenocarcinoma Transcript-1 (MALAT-1) expression [18]. MALAT-1 overexpression in NSCLC has been proposed as a poor prognostic marker that is associated with metastasis and shorter survival [19,20] in relation to EMT, cell migration, and tumor growth regulation [21,22].

In NSCLC, several studies have reported an overexpression of miR-9 in cancer tissues in comparison to normal adjacent tissues, and this overexpression has been correlated with adverse clinical features and unfavorable survival [7,23,24,25,26,27]. Some studies have also analyzed the link between miR-9 expression and treatment efficiency, showing again, contrary results depending on treatment. The growth inhibitory effect of Erlotinib was reduced with ectopic overexpression of miR-9 in adenocarcinoma cell lines [23]. On the contrary, miR-9 overexpression in the A549 (NSCLC) cell line enhanced cisplatin sensitivity by targeting the eukaryotic translation initiation factor 5A2 [28] and improved the efficiency of ionizing radiation treatment [16]. This disparity may be the result of variations in miR-9 genes’ methylation, an epigenetic mechanism, leading to transcriptional silencing. Erlotinib activates *microRNA-9-1* methylation, inhibiting its transcription [29], and radiation enhances the DNA methylation of the *microRNA-9-3* promoter [16]. Moreover, no solid concept has been established regarding the prognostic value of miR-9 genes methylation in NSCLC [23,29,30,31,32].

In a previous study, our team has explored A-type lamins (lamin A and lamin C) in metastatic lung adenocarcinoma cells from pleural effusions. Both prelamin A (precursor of lamin A) and lamin C are encoded by the *LMNA* gene. We found a reduced expression of lamin A but not of lamin C in a sub-group of patients. The reduction in lamin A expression was correlated with the loss of epithelial membrane antigen (EMA)/MUC-1, which is an epithelial marker that is involved in EMT. Moreover, the lamin A expression was inversely correlated with the number of metastatic sites and the WHO (World Health Organization) *Performance status*. Thus, we postulated that low lamin A but not lamin C expression in pleural metastatic cells could represent a major actor in the development of metastasis that is associated with EMT and could account for a pejorative factor correlated with a poor *Performance status* in lung adenocarcinoma [33].

In another context, the same expression pattern of A-type lamins has been previously described in neural cells. Two publications have showed that miR-9 is physiologically and highly expressed in these cells, in which it inhibits the expression of lamin A but not of lamin C, by directly targeting prelamin A mRNA [34,35].

Thus, in this context, miR-9 could be a good candidate to explain the reduction of lamin A expression, as miR-9 seems to be a central actor in the carcinogenesis and the metastatic process of NSCLC, and it also specifically targets prelamin A mRNA, inhibiting the expression of lamin A but not of lamin C.

## 2. Results

### 2.1. miR-9 Expression in Total Cells from Metastatic Pleural Effusions

Western blot and RT-qPCR analysis were performed from 16 pleural effusions containing metastatic cells from lung adenocarcinoma, without any cell selection.

**Figure 1:**
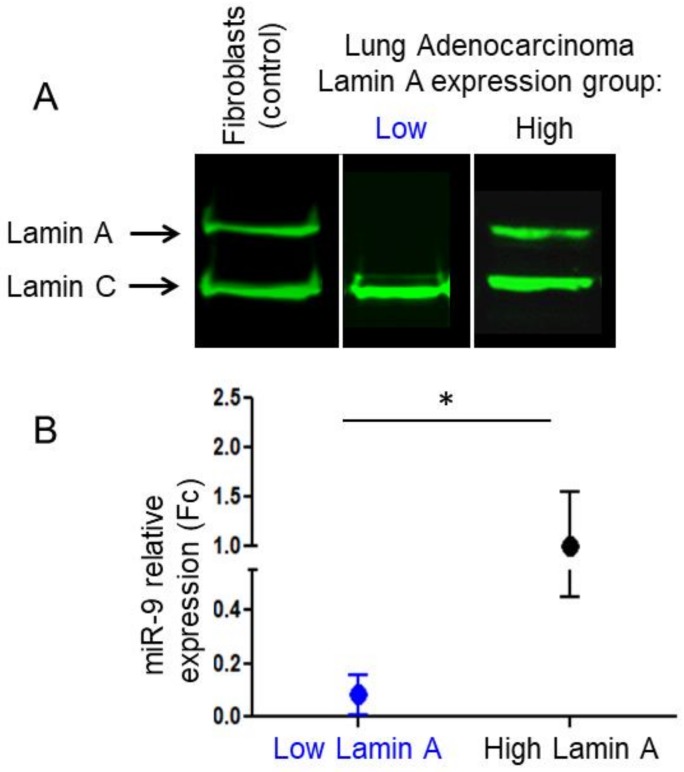
Lamin A and miR-9 expression in total cells from metastatic pleural effusions. (**A**) Representative Western blot analysis of protein extracts from patient from the ‘Low Lamin A expression’ group, from patients from the ‘High Lamin A expression’ group and from the total proteins extracts of control dermal fibroblasts using a mouse anti-lamin A/C antibody (Jol2). (**B**) miR-9 relative expression (fold change Fc) quantified by RT-qPCR analysis according to the Lamin A expression group (*p* = 0.027, Mann–Whitney). *: *p* < 0.05.

Western blot analysis showed a strong reduction in lamin A but not in lamin C expression in 7 patients, which led to classify these patients in the ‘Low lamin A expression’ group, with a ratio [Lamin A/(Lamin A + Lamin C)] < 0.2. All other patients (*n* = 9) were classified in the ‘High lamin A expression’ group as a strong lamin A expression was observed, with a ratio [Lamin A/(Lamin A + Lamin C)] ≥ 0.2 (Figure 1A) [33].

miR-9 expression was calculated using the 2^−ΔΔCT^ method, corresponding to Fold change (Fc), and was compared between pleural effusions from the 7 patients from the ‘Low lamin A expression’ group and from the 9 patients from the ‘High lamin A expression’ group [36]. Fc values were significantly lower in the ‘Low lamin A expression’ group compared to the ‘High lamin A expression’ group (*p* = 0.027, Mann–Whitney test) (Figure 1B).

These results indicate that miR-9 expression is reduced in cells from metastatic pleural effusions exhibiting low lamin A amounts.

### 2.2. Carcinoma Cells Isolation According to Lamin A Expression

As metastatic pleural effusions often contain hematopoietic reactional cells (macrophages and neutrophils) highly expressing miR-9 [37,38], we analyzed miR-9 expression specifically on carcinoma cells to confirm these results.

**Figure 2:**
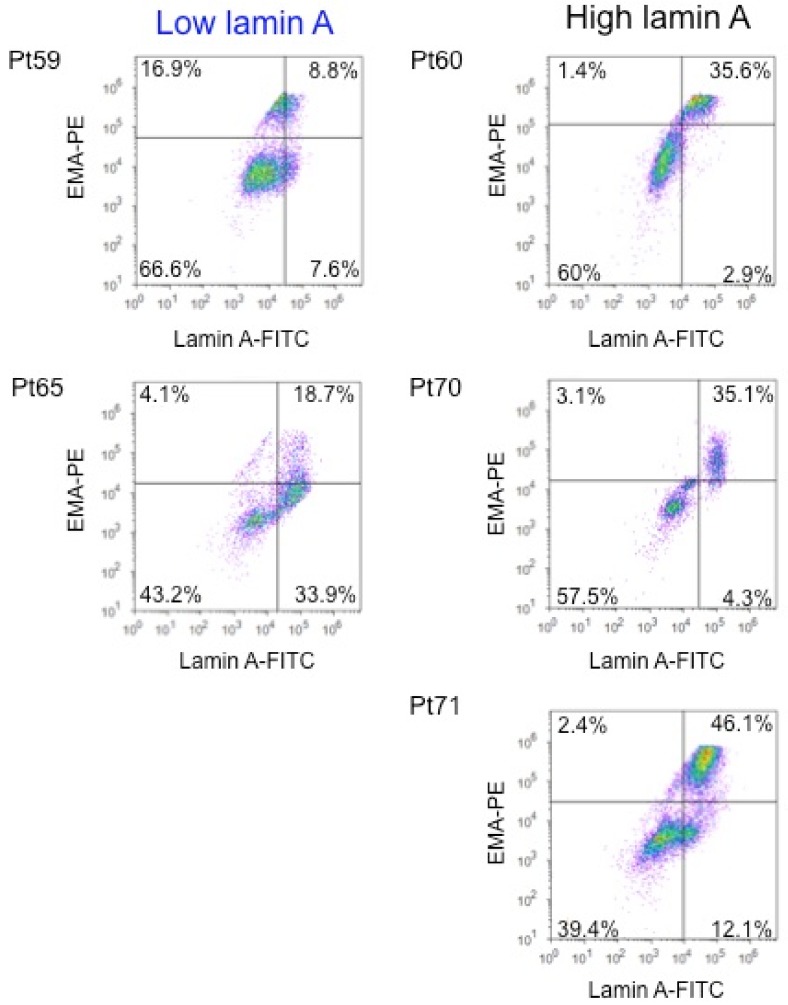
Flow cytometry analysis of lamin A and epithelial membrane antigen (EMA) in adenocarcinoma cells from pleural effusions. Representative results of lamin A and EMA expression in malignant cells contained in metastatic pleural effusions from lung adenocarcinoma (left panels = Patients (Pt) 59 and 65, from the ‘Low Lamin A expression’ group and right panels = Pt 60, 70, and 71, from the ‘High Lamin A expression’ group) using flow cytometry. Positivity thresholds were defined using isotype controls.

Thus, we performed a selection by fluorescence-activated cell sorting (FACS) of carcinoma cells from 5 patients (2 ‘Low lamin A’ patients and 3 ‘High lamin A’ patients). Leucocytes were excluded using the CD45 marker and malignant cells were selected according to EMA expression, reflecting lamin A expression. Indeed, as previously described, lamin A expression is positively correlated with epithelial membrane antigen, also known as MUC-1 (EMA/MUC-1) expression in lung adenocarcinoma cells [33] (Figure 2). Moreover, cells selection with EMA, a membrane antigen, rather than lamin A, a nuclear antigen, avoids permeabilization and fixative reagents use, which could deteriorate RNAs and compromise their extraction.

Ten samples from 5 patients were isolated, corresponding to 2 populations for each patient: EMA+ and EMA− carcinoma cells, corresponding to lamin A+ and lamin A− carcinoma cells, respectively (Figure S1). The number of sorting cells was from 8483 to 208,563 for EMA𢈒 cells and from 1213 to 184,690 for EMA+ cells.

### 2.3. miR-9 Expression, Monitored by RTq-PCR, after FACS Selection of Carcinoma Cells According to Lamin A Expression

**Figure 3:**
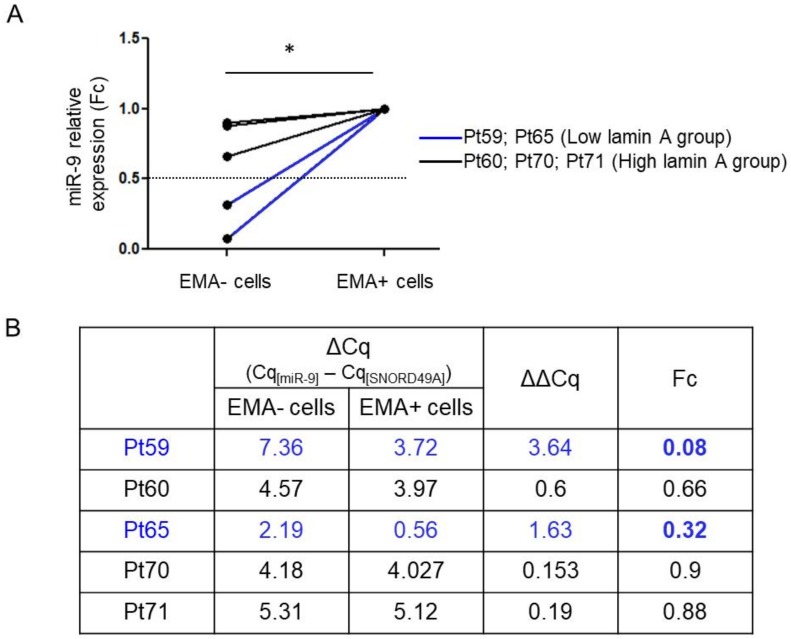
miR-9 expression in carcinoma cells according to lamin A expression, after FACS isolation. (**A**) miR-9 relative expression (Fc = fold change) quantified by RT-qPCR analysis according to the EMA expression, reflecting lamin A expression (*p* = 0.03, Paired Wilcoxon test). *: *p* < 0.05 (**B**) miR-9 relative expression and fold change (Fc) of EMA− cells compared to EMA+ cells in 2 patients from the ‘Low lamin A expression’ group (Pt59 and 65, in blue) and in 3 patients from the ‘High lamin A expression’ group (Pt60, 70 and 71, in black).

miR-9 relative expression (fold change) was calculated by the 2^−ΔΔCT^ method. Fc values were statistically lower in the EMA− cells compared to the EMA+ cells (*p* = 0.03, Paired Wilcoxon test) (Figure 3A).

Moreover, miR-9 expression was compared for each patient between EMA− and EMA+ carcinoma cells (ΔΔCq and fold change (Fc) [36]), corresponding to lamin A− and lamin A+ carcinoma cells, respectively. We observed that miR-9 was underexpressed (Fc < 0.5) in lamin A− carcinoma cells compared to lamin A+ carcinoma cells, for the two patients from the ‘Low lamin A expression’ group, whereas miR-9 was not deregulated (0.5 ≤ FC < 2) in lamin A− carcinoma cells for the 3 patients from the ‘High lamin A expression’ group (Figure 3B).

## 3. Discussion

In a previous study, our team demonstrated that the reduction of lamin A expression in pleural metastatic cells could account for a pejorative factor correlated with a poor *Performance status* in lung adenocarcinoma [33]. As miR-9 is described to directly target prelamin A (lamin A precursor) mRNA [34,35], we therefore expected that (1) lamin A expression variations were due to miR-9 in the context of metastatic cells from lung adenocarcinoma, and (2) miR-9 expression was inversely correlated with lamin A expression. Surprisingly, opposite results were obtained, as miR-9 expression was significantly reduced in patients from the ‘Low lamin A expression’ group in comparison to patients from the ‘High lamin A expression’ group. These data were also confirmed after carcinoma cells selection according to EMA expression, reflecting lamin A expression: miR-9 was underexpressed in lamin A negative carcinoma cells compared to lamin A positive carcinoma cells in patients from the ‘Low lamin A expression’ group. These observations suggest that in this context, miR-9 may not be associated with a poor prognosis, supporting the dual role of miR-9 as a tumor suppressor or an oncogene. In NSCLC, miR-9 overexpression is proposed as a pejorative marker. However, miR-9 expression has mainly or even exclusively been analyzed on lung cancer tissues from the primitive tumor, while our study has explored a metastatic site. Thus, it would be of interest to compare in a prospective study miR-9 expression in metastatic sites as well as in primitive tumor. These discordant results may also be explained by genetic and epigenetic factors regulated differently between the primary tumor and metastatic site. Little is known about the transcription regulation of the three genes encoding miR-9, depending on tissue or tumor type. Concerning epigenetic factors, miR-9 genes’ methylation frequently occurs in NSCLC primary tumors, with no obvious role of this miR-9 silencing on prognosis and tumorigenesis [29]. Lastly, several long noncoding RNAs (lncRNAs) are now described to act as ‘sponges’, inhibiting miR-9 expression, in the context of cancer or autoimmune disease [39,40,41], whatever the encoding gene.

## 4. Materials and Methods

### 4.1. Patients Samples

Sixteen patients with a pleural effusion containing metastatic cells from lung adenocarcinoma were included. This study was observational with no intervention. Pleural effusions were punctured in the Thoracic Oncology, Pleural Disease, and Interventional Pulmonology Department at Marseille North Hospital. Pleural effusions were received in the Cell Biology Laboratory at La Timone Hospital of Marseille for conventional cytological diagnosis. Using a light microscope (Leica, Wetzlar, Germany), malignant cells were manually counted, and conventional cytological analysis was completed by immunocytochemical phenotyping. The remaining fraction of the sample was used to prepare cell pellets and was stored at −70 °C until use.

The study was registered on the ClinicalTrials.gov web site (identifier: NCT01284777—27 January 2011). The protocol was approved by the Marseille Ethical Committee (Comité de Protection des Personnes Sud Méditerranée I—Reference number: 2010-A00295-34) and performed in accordance with the Declaration of Helsinki. Subjects provided informed written consent before participation.

### 4.2. Proteins Extraction from Pleural Effusion Cells

Total or nuclear proteins were extracted from frozen cell pellets.

For nuclear proteins extraction, cells were incubated during centrifugation (10 min, 800 g, 4 °C) in lysis buffer #1 (10 mM Tris HCl pH 7.5, 30 mM NaCl, 3 mM MgCl_2_, 1 mM PMSF, 0.5 μg/mL aprotinin, 0.5% NP40, and 1X Complete EDTA free protease inhibitor cocktail (Roche, Meylan, France)). Then, the supernatant was removed, and the pellet was washed and incubated for 1 h at 4 °C in lysis buffer #2 (50 mM Tris HCl pH 7.5, 250 mM sucrose, 5 mM MgSO_4_, 1 mM PMSF, 0.5 μg/mL aprotinin, 0.5% NP40, 500 kU/mL DNase (D-5025, Sigma-Aldrich, Saint-Quentin Fallavier, France), 25 kU/mL RNase (D-5503, Sigma), and 1X Complete EDTA-free protease inhibitor cocktail). After centrifugation (800 g, 15 min, 4 °C), the supernatant was removed, and the pellet was dissolved in 1.6 M NaCl.

Concerning total proteins extraction, the following lysis buffer was used: 1% Triton X100, 0.1% SDS, 0.5% sodium deoxycholate, 150 mM NaCl, 1 mM EDTA, 20 mM Tris-HCl pH 7.5, 1X Complete EDTA-free protease inhibitor cocktail, 1 mM Na_3_VO_4_, 1 mM PMSF. Cells were sonicated twice (30 s each), incubated at 4 °C for 30 min, and then centrifuged at 10,000 g for 10 min. The supernatant was frozen until use for further analysis.

Protein concentrations were determined using the BCA™ Protein Assay (Thermo Scientific, Courtaboeuf, France) according to the manufacturer’s instructions.

### 4.3. Western Blot Analysis and Lamin A Quantification

Proteins were analyzed by a standard Western blotting procedure, which was described as follows.

Protein lysates were separated on Criterion™ XT 7% Tris-Acetate precast gels (345-0135, Bio-Rad, Marnes-la-Coquette, France) and transferred to Immobilon-FL PVDF membranes (Merck-Millipore, Darmstadt, Germany). Membranes were blocked for one hour in 1:2 blocking buffer for near-infrared fluorescent Western blotting (Rockland, Limerick, PA, USA). The blocked membranes were incubated with primary antibodies for one hour at room temperature (RT) and then washed and incubated with IR-Dye-conjugated secondary antibodies for one hour at RT.

Secondary antibodies conjugated with IR-Dye 800 or 680 were used according to the manufacturer’s instructions (926-32212, 926-32214, 926-32223, 926-32224, 1/5000, LI-COR^®^ Biosciences, Homburg, Germany) [42]. Bound antibodies were detected and analyzed on an Odyssey^®^ V3.0 imaging system (LI-COR Biosciences) according to the manufacturer’s instructions.

The quantities of lamin A and C proteins were measured by fluorescence at infrared wavelengths, using the imaging system software. Lamin A expression was normalized to the total amount of lamins A and C, and a lamin ratio was established [Lamin A/(Lamin A + Lamin C)], leading to distinguish patients according lamin A expression, as previously described [33].

### 4.4. Total RNA Extraction From Pleural Effusions

Total RNAs were extracted from pleural effusions using the miRNeasy kit (Qiagen, Hilden, Germany), according to the manufacturer’s instructions. Samples were quantified by absorbance using NanoDrop DN-1000 spectrophotometer (ThermoFisher, Waltham, MA, USA). cDNA was synthesized from 10 ng of total RNA.

### 4.5. Flow Cytometry Analysis

Cells were stored in medium containing 90% FBS and 10% DMSO at –70 °C until use. Red blood cells were lysed before storage using NH_4_Cl lysis buffer. Cells were fixed in Fixation Buffer (BioLegend, San Diego, California, USA) at RT and washed prior to permeabilization at RT using the Permeabilization Buffer (BioLegend). Then, cells were incubated in Cell staining buffer (BioLegend) for 15 min and for 30 min with the following antibodies: anti-CD45-PECy7 (MHCD4512; Life Technologies), anti-EMA-PE (355604; BioLegend), and anti-laminA-FITC (ORB318755; Biorbyt, Cambridge, United Kingdom), which were diluted in the permeabilization buffer. The LIVE/DEAD^®^ Fixable Near-IR Dead Cell Stain Kit (L10119, ThermoFisher) was used to determine the cell viability.

Lamin A and EMA (epithelial membrane antigen, also known as MUC-1) protein expression was analyzed in malignant cells by flow cytometry (Attune^®^, ThermoFisher). The percentage of positive cells, median fluorescence intensity, and standard deviation (MFI ± SD) were measured in an average of 6,450 live, CD45-negative cells that were considered as carcinoma cells. Anti-IgG1k-PE (400114, Biolegend), anti-IgG1-PeCy7 (MG112, ThermoFisher), and anti-IgG3-FITC (401317, Biolegend) were used as isotype controls. As MFI of the negative cells population may vary depending on the cells’ size and autofluorescence, positivity thresholds were positioned according to the corresponding isotype control for each antibody. This strategy was applied for each patient.

### 4.6. Carcinoma Cells Selection by Cell Sorting

Tumoral cells were purified on a MOFLO Astrios EQ cell sorter (Beckman Coulter, Villepinte, France). Briefly, cells were sorted using a 70 µm nozzle, sheath fluid under 59 psi pressure, and droplet frequency around 93,000 Hz. Cells were sorted using the purify mode, with a 1.2 droplet mask.

Cells were stained with the following markers: LIVE/DEAD^®^ Fixable Near-IR Dead Cell Stain Kit (L10119, ThermoFisher), Cell Prolif Dye eFluor450 (65-0842-85; ThermoFisher), anti-CD45-PECy7 (MHCD4512; ThermoFisher), and anti-EMA-PE (355604; BioLegend).

To determine the spectral overlap and spillover matrix, monostaining was performed on compensation beads (AbC™ anti-mouse beads and ArC™ Amine Reactive Compensation Beads; ThermoFisher).

Regarding a gating strategy for cell sorting, cells were first selected on an FSC SSC plot.

Then, cells were selected as negatively stained for live dead, positive for proliferation dye, CD45-negative, and finally EMA-positive. To determine EMA-positive cells, EMA staining was observed on CD45+ cells (negative for EMA) and compensation beads.

Only bright EMA+ cells were sorted by the instrument (the gating strategy is available in Figure S1).

### 4.7. Total RNA Extraction after FACS Selection of Carcinoma Cells According to Lamin A Expression

Total RNAs were extracted from carcinoma cells isolated after FACS selection using the miRNeasy kit (Qiagen), according to the manufacturer’s instructions. As low amounts of RNAs were expected after cells selection by FACS, MS2 RNA bacteriophage (Sigma) was added as a RNA carrier in order to avoid miRNAs loss during RNA isolation [43]. As a result of MS2, extracted human RNAs could not be quantified. Thus, the same amount of EMA+ and EMA− cells (reflecting lamin A+ and lamin A− cells, respectively) was used for each patient.

cDNA was synthesized from 4 μL of total RNA.

### 4.8. miR-9 Quantification by RT-qPCR

cDNA was synthesized using miRCURY LNA^TM^ Universal RT microRNA PCR, Universal cDNA Synthesis Kit II (Qiagen), according to the manufacturer’s instructions.

The expression level of miR-9 was obtained using miRCURY LNA^TM^ Universal RT microRNA PCR, Exilent SYBR^®^ Green master mix (Qiagen). Quantitative PCR (qPCR) amplifications were performed in triplicate using the primers for hsa-miR-9-5p (YP00204513 - Qiagen) and hsa-SNORD49A (Qiagen) [44] on a LightCycler 480 (Roche Berlin, Germany). The quantification cycle (Cq) was used to calculate the relative miR-9 expression using normalization to hsa-SNORD49A (ΔCq).

miR-9 expression was compared between patients from the Low and High lamin A group, or between EMA− and EMA+ carcinoma cells, corresponding to lamin A− and lamin A+ carcinoma cells, respectively (ΔΔCq). miR-9 relative expression (fold change (Fc)) was calculated by the 2^−ΔΔCq^ method [36]. miR-9 was considered as upregulated if the fold change was ≥ 2 and down-regulated if the fold change was ≤ 0.5.

### 4.9. Statistical Analysis

Statistical analysis was performed by GraphPad Prism 5.04 (GraphPad Software, Inc. San Diego, CA, USA). Significant differences were determined using Mann–Whitney test or paired Wilcoxon test.

All statistical results were one-sided and a *p*-value < 0.05 was considered significant.

## 5. Conclusions

In conclusion, this pilot study is, to our knowledge, the first to investigate a potential link between miR-9 and lamin A expression in lung cancer. Our work suggests that miR-9 does not regulate lamin A expression in metastatic cells from lung adenocarcinoma. On the contrary, miR-9 expression was shown to be reduced in lamin A negative carcinoma cells. Thus, further investigations are needed to understand which molecular mechanisms lead to lamin A regulation in metastatic lung adenocarcinoma.

## Supplementary Materials

Supplementary Materials can be found at https://www.mdpi.com/1422-0067/21/5/1599/s1.
