# Supplementary material for: miR-9 Does Not Regulate Lamin A Expression in Metastatic Cells from Lung Adenocarcinoma
Source: Int J Mol Sci. 2020 Feb 26;21(5):1599. doi: 10.3390/ijms21051599 (PMC7084260; doi:10.3390/ijms21051599)
Supplement: Supplementary file 1 [file ijms-21-01599-s001.zip › FigureS1/Figure S1 Caption.docx]

Figure S1. Gating strategy of FACS selection. Carcinoma cells sorting according to EMA expression, reflecting lamin A expression. Cells were first selected on a FSC SSC plot. Live cells, negative for live dead, positive for proliferation dye and CD45 nedative were considered as carcinoma cells. Two carcinoma cell populations were obtained for each patient: EMA- and EMA+ carcinoma cells. Only bright EMA+ cells were sorted to increase the specificity of the sorting. To determine EMA positive cells, EMA staining was observed on CD45+ cells (negative for EMA).
