# Supplementary figures and images for: miR-9 Does Not Regulate Lamin A Expression in Metastatic Cells from Lung Adenocarcinoma
Source: Int J Mol Sci. 2020 Feb 26;21(5):1599. doi: 10.3390/ijms21051599 (PMC7084260; doi:10.3390/ijms21051599)

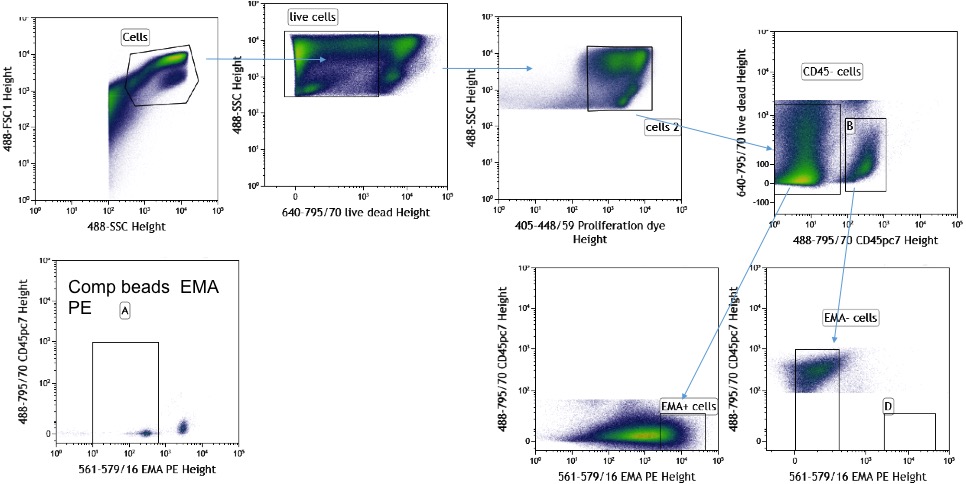

Supplement: Supplementary file 1 [file ijms-21-01599-s001.zip › FigureS1/FigS1.jpg]
